# When the Waves of European Neolithization Met: First Paleogenetic Evidence from Early Farmers in the Southern Paris Basin

**DOI:** 10.1371/journal.pone.0125521

**Published:** 2015-04-30

**Authors:** Maïté Rivollat, Fanny Mendisco, Marie-Hélène Pemonge, Audrey Safi, Didier Saint-Marc, Antoine Brémond, Christine Couture-Veschambre, Stéphane Rottier, Marie-France Deguilloux

**Affiliations:** De la Préhistoire à l'Actuel, Culture, Environnement, Anthropologie—UMR 5199, University of Bordeaux, Bordeaux, France; Natural History Museum of Denmark, University of Copenhagen, DENMARK

## Abstract

An intense debate concerning the nature and mode of Neolithic transition in Europe has long received much attention. Recent publications of paleogenetic analyses focusing on ancient European farmers from Central Europe or the Iberian Peninsula have greatly contributed to this debate, providing arguments in favor of major migrations accompanying European Neolithization and highlighting noticeable genetic differentiation between farmers associated with two archaeologically defined migration routes: the Danube valley and the Mediterranean Sea. The aim of the present study was to fill a gap with the first paleogenetic data of Neolithic settlers from a region (France) where the two great currents came into both direct and indirect contact with each other. To this end, we analyzed the Gurgy 'Les Noisats' group, an Early/Middle Neolithic necropolis in the southern part of the Paris Basin. Interestingly, the archaeological record from this region highlighted a clear cultural influence from the Danubian cultural sphere but also notes exchanges with the Mediterranean cultural area. To unravel the processes implied in these cultural exchanges, we analyzed 102 individuals and obtained the largest Neolithic mitochondrial gene pool so far (39 HVS-I mitochondrial sequences and haplogroups for 55 individuals) from a single archaeological site from the Early/Middle Neolithic period. Pairwise *F*_ST_ values, haplogroup frequencies and shared informative haplotypes were calculated and compared with ancient and modern European and Near Eastern populations. These descriptive analyses provided patterns resulting from different evolutionary scenarios; however, the archaeological data available for the region suggest that the Gurgy group was formed through equivalent genetic contributions of farmer descendants from the Danubian and Mediterranean Neolithization waves. However, these results, that would constitute the most ancient genetic evidence of admixture between farmers from both Central and Mediterranean migration routes in the European Neolithization debate, are subject to confirmation through appropriate model-based approaches.

## Introduction

The nature and mode of Neolithic expansion in Europe, also referred to as the "Neolithic Revolution", have been highly debated since the beginning of the twentieth century [1]. The Neolithic way of life, *i*.*e*., the diffusion of agriculture and farming and potentially the Neolithic people, has been shown to spread from the Near East innovation center according to two major routes of diffusion: the Continental route, following the Danube river, associated with the LBK (*LinearBandKeramik*) culture in Central Europe; and the Mediterranean route, along the Mediterranean coastlines, associated with the *Impressa* and Cardium cultures in southern Europe [2,3]. Although traditionally investigated from an archaeological standpoint, this field has substantially benefited from the study of genetics, which has notably contributed to the elaboration of a wide panel of Neolithic diffusion models through the indication of either little genetic contribution from Neolithic farmers to local hunter-gatherers (*i*.*e*., cultural diffusion) or various degrees of genetic admixture between expanding farmers and local resident groups of hunters-gatherers (*i*.*e*., leapfrog colonization or demic diffusion) [4–6]. Although genetic studies concerning extant European populations generally agree on the contribution of Neolithic farmers to the European gene pool, the degree of this contribution remains highly debated (from 20 to 70% depending on the genetic markers, the methods used and the populations targeted [6–8]). However, recent studies using appropriate model-based approaches, explicitly accounting for drift and admixture, concluded in the congruence between NRY (Non-recombining Region of the Y chromosome) and mtDNA (mitochondrial DNA) data, as both favor the demic diffusion model [9]. These authors also noted a clear decrease in the Neolithic contribution with geographic distance from the Near East (for both molecular markers). This observation suggests that both males and females admixed with the local Palaeolithic populations that inhabited Europe at that time, resulting in the progressive dilution of Near Eastern genes.

Interestingly, with recent advances in molecular biology, ancient DNA (aDNA) data have provided a deeper understanding of the processes involved in the Neolithic diffusion into Europe. Indeed, aDNA data obtained from Early and Middle Neolithic groups can provide direct insights into the gene pool of Neolithic pioneers. Thus, mtDNA data from early Neolithic groups associated with either the Danubian expansion route [10–16] or the Mediterranean route [17–19] have provided substantial evidence for (i) a turnover of mitochondrial genetic diversity with the spread of farmers from the Near East, and (ii) distinct gene pools associated with farmer groups correlated with either the Danubian or Mediterranean expansion waves. If a clear genetic discontinuity of maternal lineages at the advent of farming is regularly highlighted (genetic discontinuity between hunter-gatherers and early farmers mitochondrial lineages [10,14]), the evidence from Middle and Late Neolithic sites in Germany supports the increasing assimilation of female hunter-gatherers [11,20]. Mitochondrial data would therefore suggest a slow but steady increase in hunter-gatherer assimilation following a first period of cohabitation. Nevertheless, the emerging genome data from a few Mesolithic and early farming individuals confirms that early European farmers were primarily of Near Eastern origin and also harbored west European hunter-gatherer-related ancestry [21,22]. Then, more powerful genomic data rather suggest genetic exchange between indigenous hunter-gatherers and Neolithic communities since the advent of farmers' expansion.

The two major migration waves (*i*.*e*., Danubian and Mediterranean) may have come into contact in a region that currently encompasses the French territory [2,23]. On the one hand, the archaeological record indicates a clear cultural influence from late LBK in northeastern France, in particular in the Paris Basin (with the LBK-derived cultures RRBP *'Rubané Récent du Bassin Parisien*' and VSG *'Villeneuve-Saint-Germain*' at the end of the Early Neolithic) [24–26]. On the other hand, archaeologists have observed Mediterranean influences in the material culture found in the Paris Basin [27–30]. Although these archaeological elements demonstrate contacts occurring between Paris Basin and southern France farmer groups, they do not allow for the distinction between pure cultural exchanges versus human group gene flow. By examining the genetic relationships of populations over time, aDNA data can provide insights into the processes involved in these exchanges [14,19]. However, only three mitochondrial sequences are available for Middle Neolithic human remains from Western France [31], and no Early Neolithic aDNA data from the French territory have been published so far.

In this context, the necropolis of Gurgy 'Les Noisats' holds a key position [32]. This site is dated between 5,000 and 4,000 cal. BC, with a main occupation phase ranging from 4,900 to 4,500 cal. BC (*i*.*e*., during French Early/Middle Neolithic transition). It is localized along the Yonne River in the southern part of the Paris Basin, in the westernmost part of LBK influence (S1 Fig and S2 Fig). As opposed to the funerary monuments observed during this period in the Paris Basin [33], Gurgy is a monument-lacking necropolis grouping the burials of 128 individuals (S3 Fig). The morphology of burials could permit to draw parallels between Gurgy and western Switzerland funerary practices [34]. The homogeneity characterizing the Gurgy group (related to burial dates, funerary practices or biology; personal data) suggests that a unique population with a common cultural background used the necropolis. We genetically analyzed a total of 102 individuals to describe the maternal lineages comprising this ancient farmer group. The mitochondrial data suggested different evolutionary scenarios to explain the Gurgy gene pool make up. One scenario proposing that Gurgy necropolis reflects the genetic mixing of Neolithic farmers deriving from both Danubian and Mediterranean expansion routes finds particular resonance in archaeological data available for this region.

## Material and Methods

### Archaeological samples

Gurgy "Les Noisats" is a necropolis site in the southern Paris Basin, on the Yonne department, in Northeast France. Carbon-14 dates of human remains from this site range between 5,000 and 4,000 cal. BC (S1 Table), but the most intensive occupation period ranges from 4,900 to 4,500 cal. BC, *i*.*e*., the Early/Middle Neolithic transition in this geographic region (see S1 File for detailed archaeological context). The arrival of the "Neolithic package" in the Paris Basin, from 5,100 BC, has been associated with LBK-derived culture, called *Rubané Récent du Bassin Parisien* or RRBP [23,35–37]. Around 4,900 BC, *i*.*e*., at the end of the Early Neolithic period, a new culture called *Villeneuve-Saint-Germain* (VSG) developed in the region presenting funerary aspects clearly in the continuity of the Early Neolithic period [26]. Around 4,700 BC monumental funerary structures appeared in the Paris Basin along with the *Cerny* culture, characterized by two major types of monuments: *Structures de Type Passy* (STP) and the *Malesherbes* burials (see S1 File for details). For the period, the Gurgy necropolis corresponds to a third and more inconspicuous funerary profile observed in the region: a monument-lacking necropolis, without monument and any structuring of funerary space [33,38]. The tomb configuration discovered in the site may echo the western Switzerland and the Chamblandes cists [34]. During the same period, on the Southern part of France, the first *Impressa* settlements give way to Cardial culture around 5,500 BC [39], and from 4,900 BC the *Chasséen* culture shows an important regional diversity with a large occupation area in Southern France [40].

A total of 134 pits were excavated in the Gurgy necropolis, uncovering 128 individuals. We sampled teeth in the alveolar position, but when these samples were not available, we sampled isolated teeth or bone fragments. We acquired two samples per individual (S1 Table) for a total of 102 individuals. We followed all established aDNA guidelines to reduce contamination from the excavation site to the laboratory (S1 File). To trace the source and time of potential contamination, everyone who was in contact with the samples has been genotyped (S2 Table). Details on the authenticity of the sequences are provided in the Supporting Information. The SRA (*Service Régional de l'Archéologie*) of the Bourgogne region granted authorization for the Gurgy site archaeological excavation and sampling, with the corresponding site number, 89 198 008.

### Ancient DNA extraction

The samples were first submitted to a treatment of bleach and UV radiation. They were then reduced to powder and we followed the procedure of Mendisco *et al*. [41] to extract the DNA using the 'NucleoSpin Extract II kit' (Macherey-Nagel, Düren, Germany).

### SNP analyses

Multiplex assays targeting informative and complementary mitochondrial and Y chromosome SNPs, facilitating the characterization of common European maternal and paternal lineages, were designed with MassArray assay design software (version 4.0). Genotyping reactions were performed using the iPLEX Gold technology (Sequenom, Inc San Diego, CA, USA). The reliability and accuracy of this MALDI-TOF MS-based SNP genotyping technique were particularly adapted to the minute amount of degraded aDNA molecules [41]. A total of 28 mitochondrial SNPs and 10 Y chromosome SNPs were targeted and positive profiles were confirmed at least four times per individual (two genotyping on at least two DNA extracts per sample). The targeted SNPs and the associated primers are detailed in the S3 Table. The hierarchical structure of the targeted SNPs constitutes a powerful internal control for contamination or erroneous results.

### HVS-I Amplification and sequencing

We amplified four overlapping fragments [42] to characterize 392 bp (nps 16,009–16,400) of the mtDNA HVS-I control region (see S1 File for details on PCR amplification conditions). At least eight independent PCR reactions were performed per individual (four overlapping fragments—two extractions). All reported mutations were established according to the revised Cambridge Reference Sequence (rCRS) [43,44] and were deduced from the “consensus” among several sequences from multiple amplification products and extracts.

### Population groupings

To compare population samples with Gurgy, we distinguished sample groups based on the lower time bound of the Gurgy site occupation period (*i*.*e*., 4,000 years BC). We divided ancient hunter-gatherers (HG) and farmers (F) into six groups: European hunter-gatherers anterior to 4,000 BC ('PRE_HG'; N = 41) and European hunter-gatherers dated from after 4,000 BC ('POST_HG'; N = 30), Central European Neolithic farmers anterior to 4,000 BC ('PRE_Central_F'; N = 147) and Central European Neolithic farmers dated from after 4,000 BC ('POST_Central_F'; N = 28), Southern European Neolithic farmers anterior to 4,000 BC ('PRE_South_F'; N = 56) and Southern European Neolithic farmers dated from after 4,000 BC ('POST_South_F'; N = 49) (S4 Table). In addition, we used 20,535 modern HVS-I sequences (between nucleotide positions 16,024 and 16,380) from 78 modern populations from Europe and the Near East (S5 Table).

### Statistical Analyses

**Fig 1:**
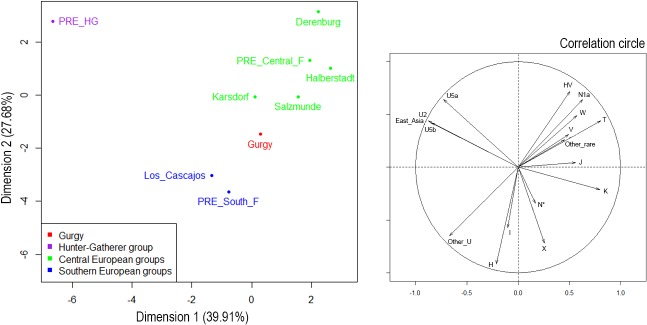
Principal Component Analysis (PCA) on the ancient mtDNA dataset. PCA performed with haplogroup frequencies and circle of correlation of the PCA. The ancient samples (S4 Table) included 'PRE_HG' for European Hunter Gatherers anterior to 4,000 BC (N = 41), 'PRE_Central_F' for Central European Neolithic farmers anterior to 4,000 BC (N = 147), and 'PRE_South_F' for Southern European Neolithic farmers anterior to 4,000 BC (N = 56). Some ancient groups anterior to 4,000 BC and sufficiently large enough for comparison at the population level are also shown (Derenburg, Halberstadt, Karsdorf, Salzmünde, from Germany [11,12,14,15]; and Los Cascajos from Spain [19]).

**Fig 2:**
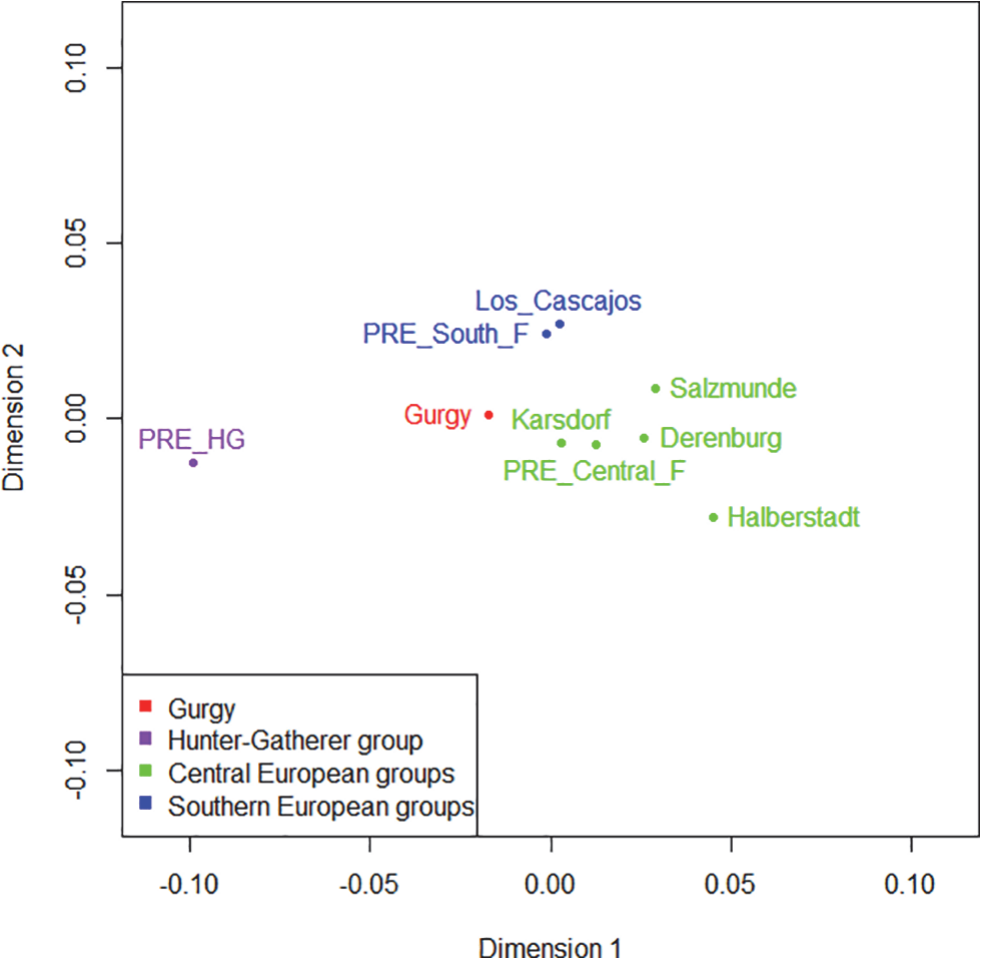
Multidimensional Scaling (MDS) on the ancient mtDNA dataset. MDS performed on the *F*_ST_ values between Gurgy and the ancient dataset (same as Fig 1).

We used Arlequin version 3.5.1.2 [45] to identify shared mtDNA haplotypes between Gurgy and other ancient and modern groups (S6 Table and S7 Table) and to compute population-specific pairwise genetic distances (*F*_ST_). The software R version 3.1.2 (Pumpkin Helmet) was used for Principal Component Analysis (Fig 1 and S4 Fig) and Multidimensional scaling (Fig 2 and S5 Fig). The shared informative haplotype frequencies were used to create a map (S6 Fig) using the software Surfer 12 (Golden Software, Inc.). A median-joining network connecting the mitochondrial sequences anterior to 4,000 BC was constructed for nps 16,056–16,380 using NETWORK 4.6.1.3. (S7 Fig).

## Results and Discussion

### Gurgy genetic diversity

From the 102 sampled Gurgy human remains, 55 mitochondrial haplogroups were generated (S8 Table), and 39 of those individuals provided HVS-I sequences (nps 16,009–16,400) that met all requested aDNA authenticity criteria (S1 Table, S1 File and S3 Fig) [46]. The sequences were deposited in the GenBank database (http://www.ncbi.nlm.nih.gov/genbank/; accession numbers KP863031-KP863069). To our knowledge, this is the largest mtDNA Neolithic sample recovered from one archaeological site for the Early/Middle Neolithic period. This sample features 27 distinct mitochondrial haplotypes belonging to eight haplogroups (H, K, U, J, N1a, V, X and T). Unfortunately, no sample yielded Y chromosome DNA, suggesting that nuclear DNA might be less well conserved in these samples. Some individuals might be closely maternally related, as five distinct mtDNA haplotypes were shared by more than two individuals (S1 Table). Only three samples sharing a haplotype (K characterized by a 16,224C mutation) originated from spatially close or even superimposed burials (GLN244, GLN245A and GLN245B). Interestingly, the archaeological data obtained for these burials showed the absence of any disturbance between different successively established burials, strongly suggesting the preserved memory of the localization of these areas.

In the last 10 years, an increasing amount of paleogenetic data has been published, providing substantial insights into the genetic relationships between ancient hunter-gatherers, ancient farmers and modern Europeans [10–19,47–62]. We used this published data (detailed in S4 Table) to examine the genetic relationships between samples from Gurgy and other temporally and spatially related aDNA samples.

The Gurgy necropolis genetic diversity (Hd = 0.9447 +/- 0.0284) is similar to the genetic diversity values obtained for Early Neolithic groups from Central Europe (e.g., Derenburg-Meerenstieg II Hd = 0.9567 +/- 0.0238 [14,15] and Halberstadt-Sonntagsfeld Hd = 0.9613 +/- 0.0178 [11,12]) but is slightly higher than that of the Los Cascajos group in Southern Europe (Hd = 0.8234 +/- 0.0670 [19]) (S9 Table). The Gurgy group shows nucleotide diversity (π = 0.010056 +/- 0.005795) intermediate between the values observed for LBK groups and farmers from southwestern Europe (S9 Table). We also observed that the Gurgy genetic diversity is close to the value calculated for the hunter-gatherer group PRE_HG (Hd = 0.9305 +/- 0.0250). This result is striking, as we observe analogous genetic diversity between a hunter-gatherer sample geographically (from Spain to Russia; S4 Table) and chronologically (from 38,000 BP to 4,450 BC; S4 Table) largely dispersed and a local group of Neolithic farmers who buried their dead in the Gurgy necropolis for approximately 500 years. This observation is consistent with population-genomic evidence suggesting lower diversity for hunter-gatherers than for farmers [21,22,63].

### Relationship of Gurgy farmers with ancient European groups

**Fig 3:**
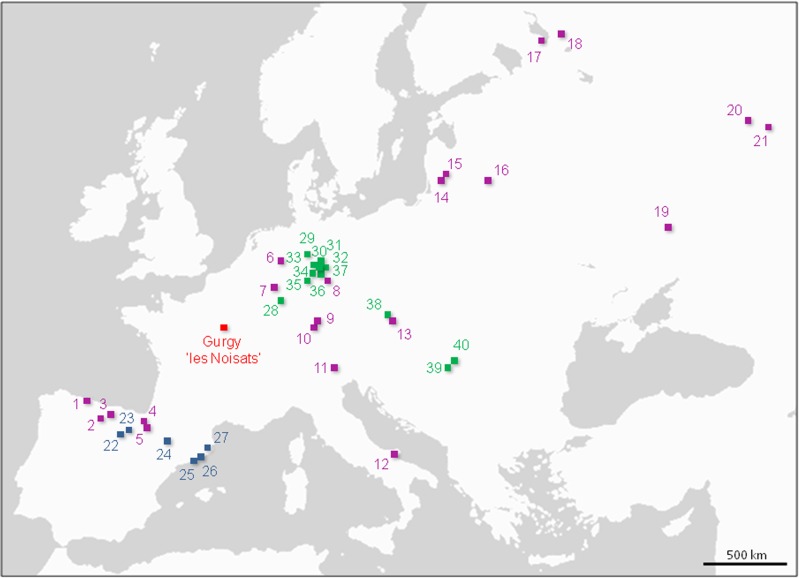
Location of the sites anterior to 4000 cal BC. **PRE_HG** (purple): 1) La Braña, 2) La Pasiega, 3) La Chora, 4) Erralla, 5) Aizpea, 6) Oberkassel, 7) Reuland-Loschbour, 8) Bad Durrenberg, 9) Hohleinstein, 10) Hohler Fels, 11) Villabruna, 12) Paglicci, 13) Dolni Vestonice, 14) Spiginas, 15) Donkalnis, 16) Kretuonas, 17) Uznyi Oleni Ostrov, 18) Popovo, 19) Kostenki, 20) Chekalino, 21) Lebyazhinka. **PRE_South_F** (blue): 22) Los Cascajos, 23) Paternanbidea, 24) Chaves, 25) Can Sadurni, 26) Sant Pau del Camp, 27) Avellaner. **PRE_Central_F** (green): 28) Flomborn, 29) Wittmar, 30) Oberwiederstedt, 31) Unterwiederstedt, 32) Salzmünde, 33) Derenburg, 34) Halberstadt, 35) Naumburg, 36) Karsdorf, 37) Esperstedt, 38) Vedrovice, 39) Szarvas, 40) Ecsegfalva. References in S4 Table.

To understand of the genetic relationships between the Gurgy group and other temporally and spatially related aDNA samples of hunter-gatherers and farmers, we divided published aDNA data into three groups anterior or contemporaneous to the Gurgy group (*i*.*e*., anterior to 4,000 BC, the most recent date obtained for Gurgy sample remains): European hunter-gatherers anterior to 4,000 BC ('PRE_HG'; N = 41), Central European Neolithic farmers anterior to 4,000 BC ('PRE_Central_F'; N = 147) and Southern European Neolithic farmers anterior to 4,000 BC ('PRE_South_F'; N = 56) (Fig 3 and S4 Table). Four common basal mtDNA haplotypes were shared between Gurgy, 'PRE_HG', 'PRE_Central_F' and 'PRE_South_F' groups (S6 Table). Three other haplotypes were shared between 'PRE_Central_F' and 'PRE_South_F' populations, and 20 haplotypes were specific to Gurgy farmers. The number of unique haplotypes encountered in the Gurgy group (similar to the Central and South European farmer groups) must be associated with the still weak aDNA dataset available for ancient European populations. A median-joining network connecting all ancient HVS-I sequences (from hunter-gatherers and farmers anterior to 4,000 BC) is proposed in S7 Fig. The resulting network is not completely satisfactory, as it only considers short HVS-I sequences, as responsible for creating artificial links between different haplogroups. Nevertheless, this network suggests that most of the Gurgy haplotypes are either shared with other ancient European farmers or likely derived from the sequences obtained from Central and South Europe farmers (S7 Fig).

**Fig 4:**
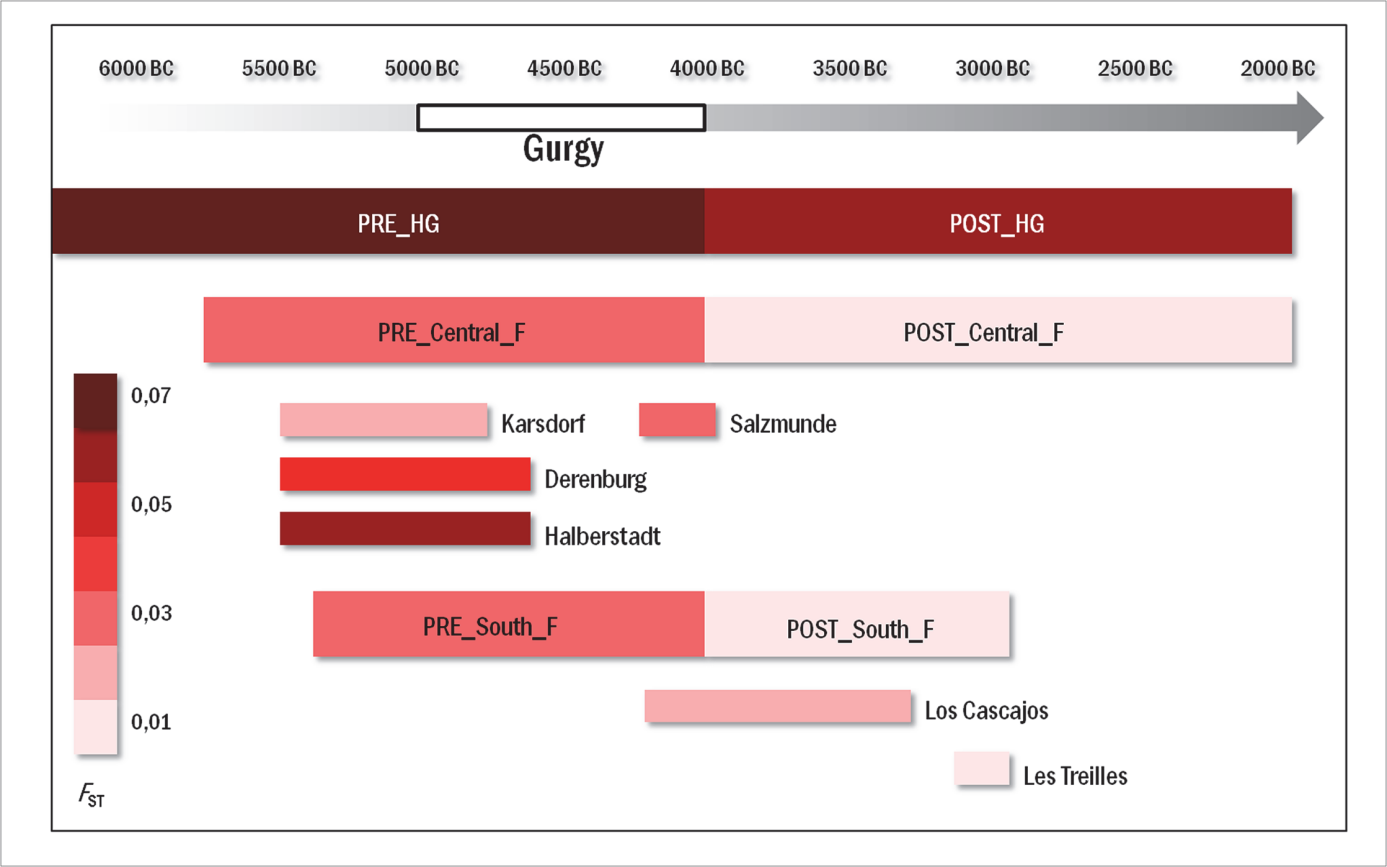
Pairwise *F*_ST_ distances. *F*_ST_ measured between Gurgy and ancient European hunter-gatherers / farmer groups (S4 Table and S10 Table). 'PRE_HG' for European hunter-gatherers anterior to 4,000 BC (N = 41), 'POST_HG' for European hunter-gatherers after 4,000 BC (N = 30), 'PRE_Central_F' for Central European Neolithic farmers anterior to 4,000 BC (N = 147), 'POST_Central_F' for Central European Neolithic farmers after 4,000 BC (N = 28), 'PRE_South_F' for Southern European Neolithic farmers anterior to 4,000 BC (N = 56), and 'POST_South_F' for Southern European Neolithic farmers after 4,000 BC (N = 49). Ancient groups anterior to 4,000 BC, which were sufficiently large for comparison at the population level are also shown (Derenburg, Halberstadt, Karsdorf, Salzmünde, from Germany [11,12,14,15]; Los Cascajos from Spain [19] and Les Treilles form southern France [54]).

Previous work has demonstrated the mtDNA distinctiveness of ancient hunter-gatherers (*i*.*e*., 'PRE_HG') compared with Early/Middle Neolithic farmers [10,11,14,19]. This point is clearly illustrated via principal component analysis (PCA) and Multidimensional Scaling (MDS) performed on the ancient groups (Figs 1 and 2). Indeed, the PCA revealed that most of the variation in mtDNA haplogroup frequencies lied between ancient hunter-gatherers (PRE_HG) and farmer groups, including Gurgy (first component, Fig 1). Notably, 'PRE_HG' is characterized by specific haplogroups, such as U2, U4, U5 and several H, which are absent or rare in Gurgy or other farmer groups. Calculation of the pairwise *F*_ST_ values between ancient populations confirmed an important genetic distance between ancient European hunter-gatherers and Gurgy farmers (0.08016; Fig 2 and Fig 4 and S10 Table). These observations are consistent with the results from Bramanti *et al*. [10] and Haak *et al*. [14], who proposed the genetic discontinuity of maternal lineages between local hunter-gatherers and early farmers (a genetic distinction that decreased during later periods through progressive hunter-gatherers assimilation [11]). Notably, the *F*_ST_ value measured between Gurgy and PRE_HG groups was slightly lower than that measured between other ancient farmers groups (from Central and southern Europe) and PRE_HG. These results are consistent with the study of Rasteiro and Chikhi [9], showing a clear decrease in the Neolithic contribution / higher hunter-gatherer admixture with geographic distance from the Near East. The involvement of hunter-gatherers in the constitution of the Gurgy gene pool requires more powerful model-based approaches for further clarification. However, the important genetic distance measured between Gurgy and hunter-gatherer groups clearly contrasts with the genetic similarities observed between Gurgy and Early Neolithic farmers from Central and Southern Europe.

The *F*_ST_ values calculated between Gurgy and (i) the 'PRE_Central_F' and (ii) the 'PRE_South_F' groups are almost perfectly equal (0.03333 and 0.03176, respectively) and much lower than the *F*_ST_ between Gurgy and the 'PRE_HG' group (Fig 4 and S10 Table). We also computed *F*_ST_ between Gurgy and ancient groups sufficiently large enough for comparison at the population level (specific archaeological sites providing more than 20 HVS-I sequences) (Fig 4). Interestingly, low *F*_ST_ values suggest similar genetic proximity between Gurgy and some LBK populations in Germany (Karsdorf, Salzmünde [11,12]) or Los Cascajos in Spain [19]). The MDS constructed using the *F*_ST_ value reported above places the Gurgy group in an intermediate position between southern and Central farmer groups (Fig 2).

The PCA presented above highlights another substantial degree of variation distinguishing 'PRE_Central_F' and 'PRE_South_F', following the two major migration waves associated with Neolithic diffusion in Europe (Fig 1). In the PCA, the population of Gurgy that shares haplogroups with both Southern and Central farmer groups also revealed an expected intermediate position. Notably, Gurgy showed three individuals carrying mutations characteristic of the N1a haplogroup, which is considered a genetic marker of the Danubian expansion route because it is common among Early Neolithic farmers from Central Europe (14 N1a sequences are currently associated with LBK groups [11,14,15]) and has not been detected in farmers groups associated with the Mediterranean migration route so far. Although the three N1a sequences from the Gurgy group differ from those reported for the LBK groups, these findings might support the implication of LBK descendants in Gurgy group formation.

In summary, all descriptive analyses highlight strong genetic differentiation between Gurgy and European hunter-gatherers and even stronger and equal genetic affinities between Gurgy and ancient farmers from both Central and South Europe. These observations might reflect distinct evolutionary scenarios such as (i) a tree-like population history with three ancestral farmer populations leading to the three farmer groups analyzed (Gurgy/Danubian farmers/Mediterranean farmers) or (ii) admixture, *i*.*e*., the Gurgy gene pool was likely shaped by a similar contribution of farmers originating from the Mediterranean region and Central Europe associated with the Danubian and Mediterranean Neolithization routes. Although the descriptive analyses did not favor any evolutionary scenario (patterns might be affected by sample composition and interpretations in terms of admixture events were not straightforward), the available archaeological data for this region support the admixture model.

### Concordant archaeological and paleogenetic clues of gene flow between Early/Middle Neolithic Paris Basin and southern France farmers

Gurgy is localized in the southern area of the Paris Basin, which lies at the westernmost part of the LBK cultural influence (S1 Fig and S2 Fig) [2,24]. The Gurgy necropolis shows a potential RRBP cultural tradition (clearly LBK derived) in terms of burial type, position or orientation (S1 File) [64]. However, some archaeologists have observed parallels with western Switzerland [34] and demonstrated cultural exchanges between Paris Basin populations and groups from southern France associated with Cardium and *Chasséen* cultures [27,28,30,65]. Various artifacts or cultural influences from southern France, corresponding to pottery decorations [27], bone industry [29,66] or personal ornaments (*Spondylus* shells or white limestone rings [67,68]), have been observed in the RRBP or Villeneuve-Saint-Germain (VSG) burials of the Paris Basin. Two hypotheses have been proposed to explain this southern cultural influence in farmer groups from the Paris Basin, including either an appropriation of techniques and style through genetic contact and admixture between populations or a simple exchange of artifacts and raw materials without gene flow [29]. We propose that the archaeological data and the paleogenetic results obtained on the Gurgy necropolis are consistent with the hypothesis that the Mediterranean cultural influence on the Paris Basin entails some gene flow, implying the northerly migration of Mediterranean farmers in the Paris Basin and admixture with farmer descendants of LBK populations from Central Europe. As proposed by Sidéra [29,69], exogamy between groups might explain the fusion of the material culture of both Central and southern Neolithic spheres in the Paris Basin and the mixture of southern/Central Europe farmer lineages observed in the Gurgy necropolis.

### Gurgy and the evolution of farmer groups during the Middle/Late Neolithic period

To unravel the genetic affinities of the Gurgy group with later European groups, the Gurgy maternal gene pool was compared with that of hunter-gatherer or farmer populations dated between 4,000 BC and 2,000 BC: European hunter-gatherers after 4,000 BC ('POST_HG'; N = 30), Central European Neolithic farmers after 4,000 BC ('POST_Central_F'; N = 28), and southern European Neolithic farmers after 4,000 BC ('POST_South_F'; N = 49) (S4 Table and Fig 3). Gurgy mtDNA diversity showed clear genetic affinities (in term of lowest *F*_ST_) with 'POST_Central_F' (0.00965), 'POST_South_F' (0,01774), or the French group of Les Treilles (0.01006; southern France, 3,000 BC [54]). Paleogenetic data for the Middle or Late Neolithic periods indicated that migratory/demographic events have largely remodeled the gene pool of farmer groups throughout the Neolithic period. This evidence was recently supported by an analysis of a time transect spanning the >3,500 years of the Central European Neolithic period highlighting substantial gene flow throughout the European continent during the Late Neolithic period [11]. Consequently, farmer groups from the Late Neolithic period were formed from a mosaic of maternal lineages from diverse European origins, and this mixed constitution might explain the genetic similarity of these farmer groups to Gurgy.

### The relationship of Gurgy farmers with extant European populations

The Gurgy mitochondrial gene pool wascompared with 20,535 HVS-I sequences compiled from 78 European and Near Eastern modern populations to assess the similarity between the Gurgy and modern European gene pools (S4 Table and S5 Fig). PCA revealed genetic differentiation between modern populations from the Near East and those from Europe (S4 Fig) and high genetic homogeneity of modern European populations. Interestingly, the Gurgy group was isolated from the both blocks, suggesting that global European gene pool remodeling since the Neolithic period contributed to the observed discontinuity between early Neolithic groups (including Gurgy) and modern European mitochondrial gene pools [11,12,14,15,70].

We next assessed haplotypes shared between the Gurgy and modern European populations. Excluding the four common and non-informative haplotypes observed in Gurgy and a large number of European populations (mainly basal haplotypes) and excluding the eleven haplotypes unique to Gurgy, twelve remaining informative haplotypes were shared between Gurgy and specific European and Near East populations (S7 Table). We considered that these informative haplotypes might potentially highlight extant populations with affinities to the Gurgy group, presumably representing a genetic legacy from the Neolithic period [14]. The map specifically reporting these shared informative haplotype frequencies shows interesting hotspots in Turkey and the Balkan Peninsula and along Danube Valley and the Mediterranean shores (S6 Fig). Thus, the geographic distribution of these informative haplotypes in modern European populations might reflect some aspects of alleged Gurgy group history, *i*.*e*., the scenario proposing that the group comprised an admixture between the descendants of the farmers from Danubian and Mediterranean expansion routes, who are the descendants of farmers from the Balkans and Anatolia.

## Conclusion

We conducted a paleogenetic study on the Gurgy 'Les Noisats' necropolis, presenting the first genetic data on Neolithic settlers (Early/Middle Neolithic transition) in France. Localized in the southern part of the Paris Basin, the necropolis provides a unique opportunity to document the processes implied in the Neolithization of a region where cultural exchanges between culturally distinct farmer groups have been highlighted. Descriptive analyses performed using Gurgy mtDNA diversity highlight a gene pool clearly intermediate between those characterized for the farmers associated with both Danubian and Mediterranean migration routes. Even if these findings were consistent with different evolutionary scenarios, we propose that the scenario in which the Gurgy gene pool resulted from equivalent contributions of maternal lineages from farmer groups associated with the Danubian and Mediterranean expansion routes is the most parsimonious. These arguments notably corroborate archaeological evidence of cultural exchanges between farmers from the Paris Basin and those from southern France, indicating that the observed cultural exchanges reflect genetic admixture between groups. However, we are fully aware that these data do not definitively prove admixture between farmers associated with both Neolithization routes and that the proposed admixture model must be tested under a robust analytical framework, such as the coalescent theory that would account for genetic drift and population demography within a gene genealogy [71].

## Supporting Information

S1 Fig Simplified map of Neolithic cultures expansion in France concerning the Paris basin during the end of the Early Neolithic period (5800–4600 BC). (TIF)

S2 Fig Simplified map of Neolithic cultures expansion in France concerning the Paris basin during the beginning of the Middle Neolithic period (4700–4300 BC). (TIF)

S3 Fig Map of Gurgy necropolis with characterized haplogroups and haplotypes. (TIF)

S4 Fig Multidimensional Scaling Analysis (MDS). Modern dataset (S5 Table).
(TIF)

S5 Fig Principal Component Analysis (PCA). Modern dataset (S5 Table).
(TIF)

S6 Fig Shared informative haplotypes. Map featuring the frequency distribution of informative haplotypes shared between Gurgy and modern populations (S5 Table).
(TIF)

S7 Fig Median-joining Network. Performed using the HVS-I sequences (nps 16,056–16,380) available for hunter-gatherers and farmers anterior to 4,000 BC (S4 Table).
(EPS)

S1 File Material and Methods. (DOCX)

S1 Table Gurgy aDNA results. (XLSX)

S2 Table Manipulators genetic data. (XLSX)

S3 Table SNP primers. (XLSX)

S4 Table Population references for the ancient dataset. (XLSX)

S5 Table Population references for the modern dataset. (XLSX)

S6 Table Shared haplotype frequencies for the ancient dataset. (XLSX)

S7 Table Shared haplotype frequencies for the modern dataset. (XLSX)

S8 Table Detailed mitochondrial SNPs obtained for positive Gurgy individuals. (XLSX)

S9 Table Genetic diversity indices for the ancient dataset. (XLSX)

S10 Table Matrix of *F*_ST_ values. (XLSX)

S11 Table Modern dataset references for the shared haplotype frequencies table (S7 Table). (XLSX)

S12 Table Genetic diversity indices for the modern dataset. (XLSX)
