# Supplementary figures and images for: When the Waves of European Neolithization Met: First Paleogenetic Evidence from Early Farmers in the Southern Paris Basin
Source: PLoS One. 2015 Apr 30;10(4):e0125521. doi: 10.1371/journal.pone.0125521 (PMC4415815; doi:10.1371/journal.pone.0125521)

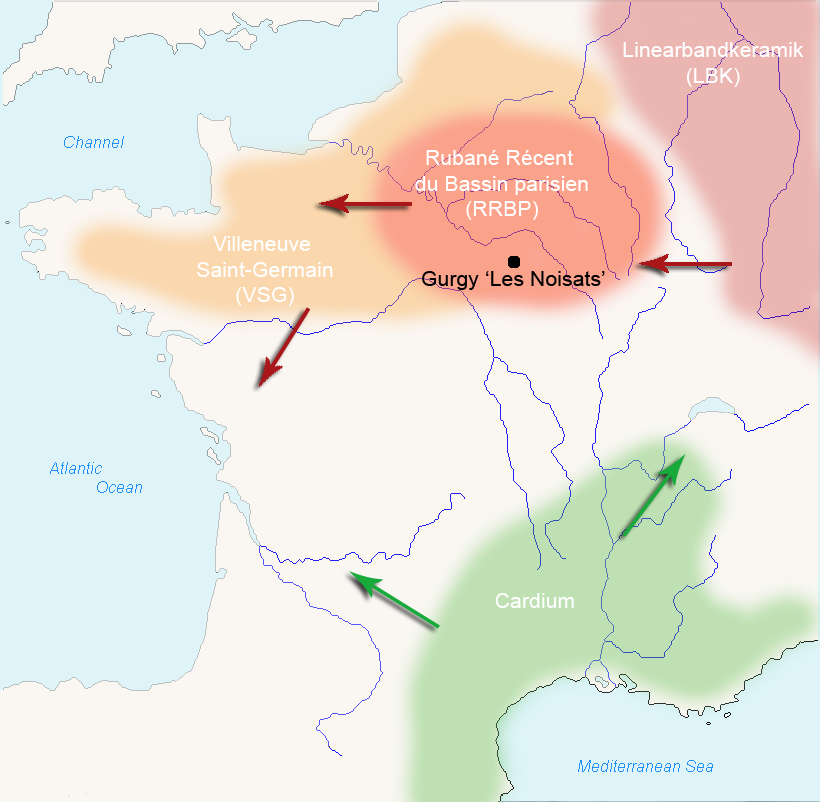

Supplement: S1 Fig — (TIF) [file pone.0125521.s001.tif]

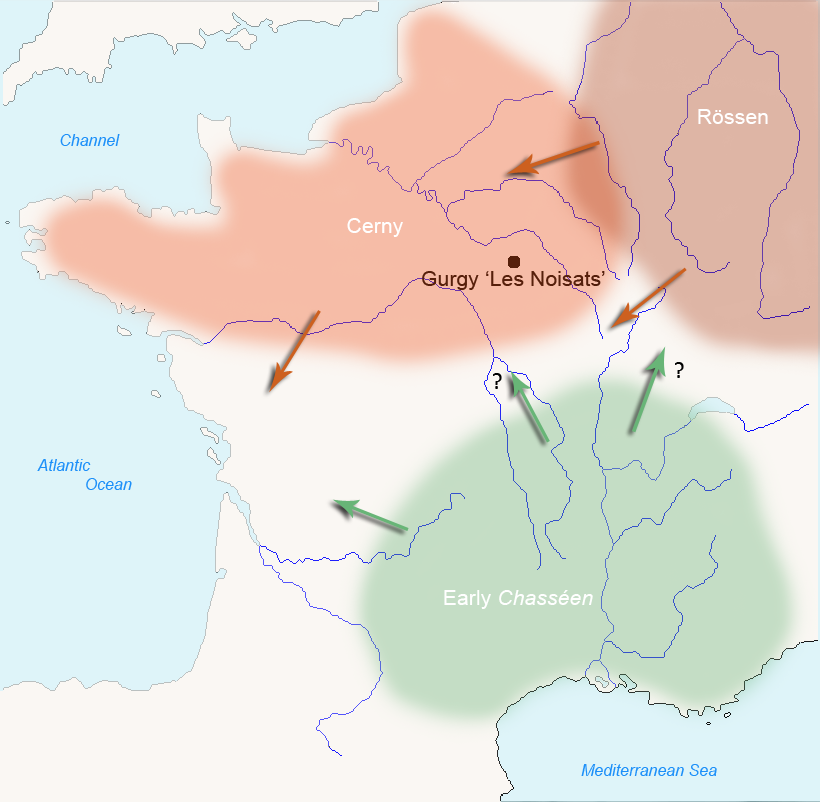

Supplement: S2 Fig — (TIF) [file pone.0125521.s002.tif]

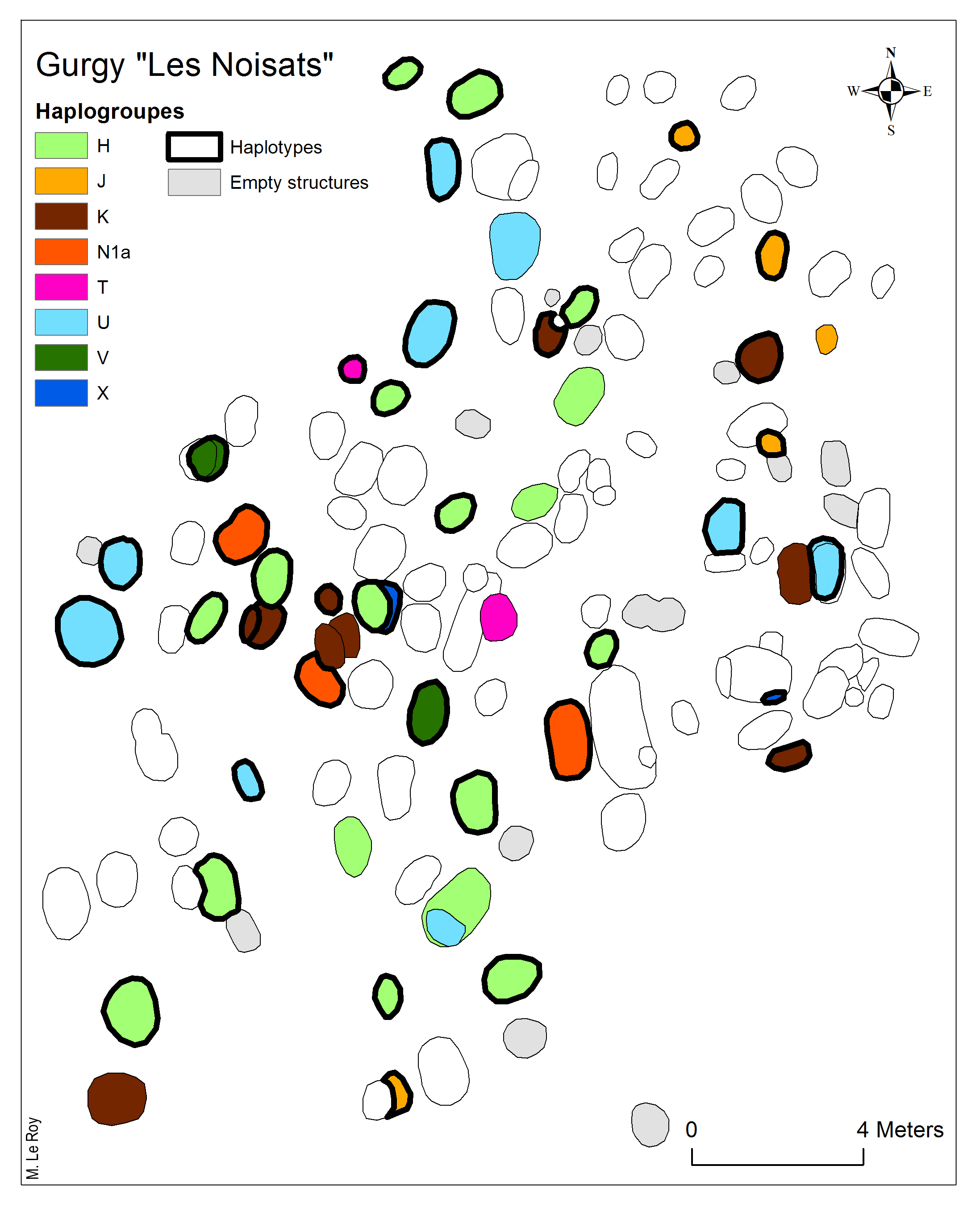

Supplement: S3 Fig — (TIF) [file pone.0125521.s003.tif]

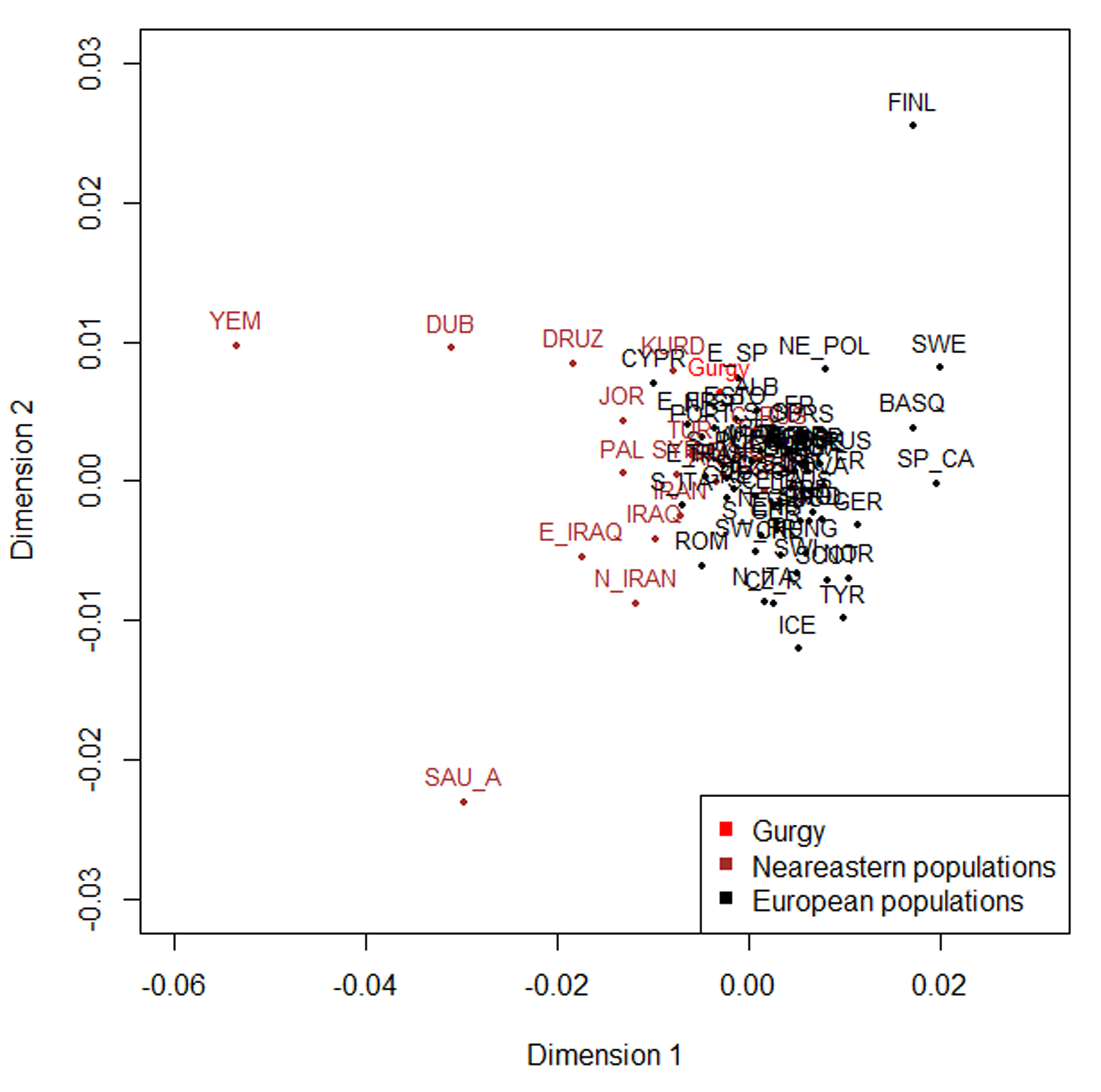

Supplement: S4 Fig — Modern dataset (S5 Table). (TIF) [file pone.0125521.s004.tif]

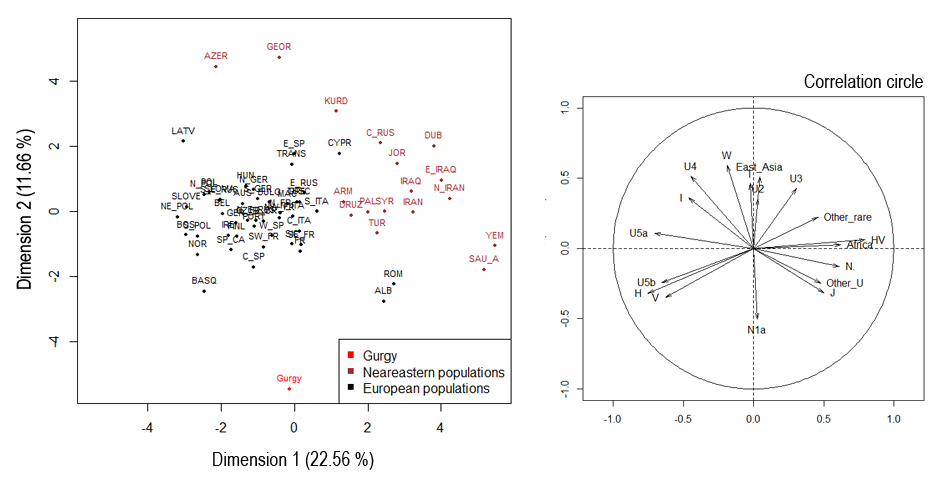

Supplement: S5 Fig — Modern dataset (S5 Table). (TIF) [file pone.0125521.s005.tif]

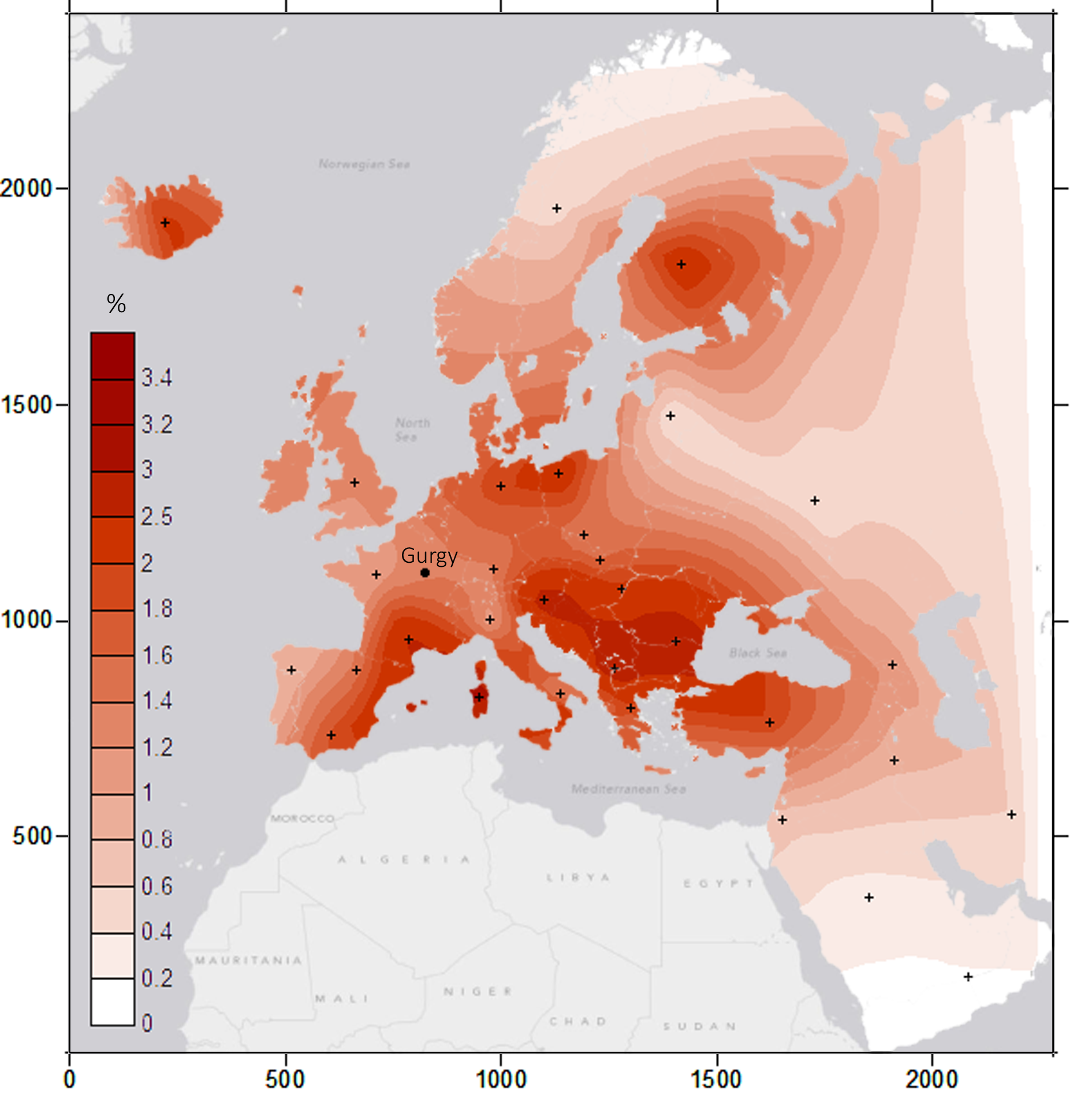

Supplement: S6 Fig — Map featuring the frequency distribution of informative haplotypes shared between Gurgy and modern populations (S5 Table). (TIF) [file pone.0125521.s006.tif]
