# Supplementary material for: When the Waves of European Neolithization Met: First Paleogenetic Evidence from Early Farmers in the Southern Paris Basin
Source: PLoS One. 2015 Apr 30;10(4):e0125521. doi: 10.1371/journal.pone.0125521 (PMC4415815; doi:10.1371/journal.pone.0125521)
Supplement: S1 File — (DOCX) [file pone.0125521.s008.docx]

**When the waves of European Neolithization met: first paleogenetic evidence from early farmers in the southern Paris Basin.**

Maïté Rivollat, Fanny Mendisco, Marie-Hélène Pemonge, Audrey Safi, Didier Saint-Marc, Antoine Brémond, Christine Couture-Veschambre, Stéphane Rottier, Marie-France Deguilloux

**Supporting Information**

**Material and Methods**

**Archaeological site**

The archaeological site of Gurgy 'Les Noisats' is located in the department of Yonne, in the southern part of the Paris Basin, France [[1](#_ENREF_1)] (S1 Figure and S2 Figure). This area is characterized by an abundance of archaeological sites, particularly from prehistoric periods. Gurgy 'Les Noisats' is a necropolis excavated from 2004 to 2007. We performed ^14^C dating on 28 human remains, showing that the necropolis was used from 5,000 to 4,000 cal. BC, with a more intense occupation from 4,900 to 4,500 cal. BC (*i.e.,* during French Early/Middle Neolithic transition). From 134 thickly distributed pits, we excavated 128 human skeletons. The skeletons were mainly laid on the left side with limbs hyper-flexed, and in most cases, these remains were oriented North-South.

**Archaeological context (S1 Figure and S2 Figure)**

To understand the specific cultural features of Gurgy 'les Noisats', it is essential to describe archaeological cultures spatially connected and older and more recent to the Gurgy site, starting with the beginning of the Neolithic period in this region. Mesolithic hunter-gatherers occupied the region currently known as France when Early Neolithic settlers, associated with the *Impressa* ceramic culture, moved to the Mediterranean coast around 5,800 BC [[2](#_ENREF_2),[3](#_ENREF_3)]. This period marks the emergence of the new subsistence strategy in western Europe and corresponds to the beginning of the Cardial pottery culture in this geographic region (*i.e.,* France). Autochthonous groups from the easternmost French regions, which adopted ceramic traditions from southern France, are described as (i) *La Hoguette* in Northern Switzerland and southwestern Germany and (ii) *Limbourg* in Belgium, northeastern France and the Paris Basin [[4](#_ENREF_4),[5](#_ENREF_5)]. Around 5,200 BC, Early Neolithic *Linearbandkeramik* culture (LBK) from Central Europe arrived in the Paris Basin with cultural specificities [[6](#_ENREF_6)]. From 5,100 BC, an LBK-derived culture, called *Rubané Récent du Bassin Parisien* or RRBP [[7](#_ENREF_7),[8](#_ENREF_8)], appeared in this region. Although the RRBP presents many characteristics similar to the LBK culture (with rectangular houses, standard simple burials with bodies in contracted position), some instances of more complex burial structures with a step and an *alcove* (kind of shelter) have been observed in Aisne valley [[9](#_ENREF_9)]. The end of the Early Neolithic period in the Paris Basin, around 4,900 BC, is characterized by the development of a new culture called *Villeneuve-Saint-Germain* (VSG) [[10](#_ENREF_10)]. VSG funerary aspects were clearly in the continuity of Early Neolithic tradition with bodies observed in pits, lying on the left side in a contracted position with the head facing eastward [[11](#_ENREF_11)].

Around 4,700 BC monumental funerary structures appeared in the Paris Basin, concomitant with the *Cerny* culture, resulting in two major types of monuments. The first type of monument is called *Structures de Type Passy* (STP), associated with long monuments with an axial symmetry in an east-west orientation [[12](#_ENREF_12),[13](#_ENREF_13)]. Between two and ten burials per monument were observed, where the burial pits were situated on an axis or in a peripheral position, and the bodies were laid on their backs in a coffin. The other main funerary tendency observed during this period was *Malesherbes* burials, comprising individual burials sealed with a megalithic slab on the ground level [[14](#_ENREF_14)]. The bodies were buried in a contracted position with the head facing eastward. Despite differences between these two types of burials, these monuments partially overlapped spatially (STP being more frequent in the East of the Paris Basin, while *Malesherbes* is more frequent in the West). The without-monument necropolis is a third and more inconspicuous type of funerary profile observed in the Paris Basin, without monument and any structuring of funerary space [[15](#_ENREF_15)].

In the southern part of France, the first *Impressa* settlements gave way to Cardial culture around 5,500 BC [[2](#_ENREF_2)]. These groups were not associated with previous communities and are characterized by a different pottery style with shell-made decorations (*cardium*). The wide area of the Cardial culture distribution ranges from Italy to southern Spain along Mediterranean coasts. From 4,900 BC, the *Chasséen* culture showed an important regional diversity and occupied a large part of Southern France [[16](#_ENREF_16)].

Gurgy 'Les Noisats' occupation was initiated at the end of Early Neolithic period and ended during the Middle Neolithic period, in the culturally rich and complex area described above. Despite the cultural diversity of this region, the site practices are rather homogeneous. Gurgy is a necropolis, without any monumental structure, with single (most frequent) and double pits (rarer), where decay in a void was observed. Wooden coffins are noteworthy for a number of burials [[17](#_ENREF_17)], and could be compared to the Chamblandes cists in western Switzerland [[18](#_ENREF_18)]. Some burials showed *alcove* features similar to those observed during the RRBP [[1](#_ENREF_1),[9](#_ENREF_9)]. Few grave goods were observed in Gurgy burials, such as fauna (bones, teeth, shells), flint, ochre and rare pottery.

**Sampling**

We sampled the teeth or bones from the best 102 well-preserved individuals. Because the paleogenetic project of Gurgy 'Les Noisats' was set up during the second year of the site excavation, the burials discovered during the first year were excavated without any specific aDNA care (N=31). Stored in an archaeological warehouse since discovery, the burials were subsequently sampled in 2012 using gloves and masks, and benefited from the specific processing of decontamination (see details below). The other samples (N=71) were collected with all precautions to avoid contamination. Complete or fragmented mandibles, isolated teeth or bone fragments were sampled using gloves and masks and directly deposited in sterile plastic bags, without washing or examination. The samples were immediately stored at -20°C. Excavators in contact with the samples were genotyped (S2 Table), and manipulation traceability was used to trace potential contamination for each sample (S1 Table).

**Ancient DNA (aDNA) laboratory conditions**

All the genetic analyses were performed in the ancient DNA facilities of the Laboratory of Past Human Populations, UMR PACEA (Université de Bordeaux, Bordeaux, France) in a laboratory dedicated to analyses of aDNA. Pre-PCR procedures were conducted under sterile conditions in an aDNA room (characterized by a high-pressure system, ﬁltered incoming air, UV light irradiation, bleach cleaning of every surface, and laminar ﬂow hood), and the laboratory equipment and reagents were DNA-free. The workers systematically wore clean-room suits, shoe protection, facemasks, and gloves. DNA isolation, PCR reagent preparation and mixing and DNA extraction were conducted in three distinct rooms of the aDNA-dedicated laboratory. These rooms were spatially distant from the main laboratory, where PCR ampliﬁcation and subsequent procedures are conducted (at opposite ends of the same building) to avoid contamination from ampliﬁed DNA.

**Sample preparation and DNA extraction**

The teeth were isolated from the mandible in a sterile laminar flow hood. The 31 samples excavated without specific aDNA care were first scraped, cleaned with bleach and subsequently exposed to UV radiation for 20 minutes on each side. The 71 additional remains (supposed non-contaminated) were exposed to UV radiation for 20 minutes on each side. The DNA was extracted using the 'NucleoSpin® Extract II kit' (Macherey-Nagel, Düren, Germany) as previously described [[19](#_ENREF_19)], except for the lysis buffer. Each sample was preliminarily reduced to powder and incubated in a lysis buffer (0.5 M EDTA, pH 8, 25 mg/mL proteinase K, and 0.5% *N*-Lauryl sarkosyl) overnight at 55°C. The DNA extraction was performed according to the manufacturer's instructions, and 80 µL was concentrated in Amicon® columns and stored at -20°C.

**SNP multiplex design**

We performed a multiplex SNP typing designed using MassArray assay design software (version 4.0) to assess DNA conservation for every sample. A total of 28 mitochondrial SNPs and 10 Y chromosome SNPs were targeted. These SNPs facilitated the characterization of the occurrence of specific mutations for given haplogroups. The synthetic primers used for these experiments are listed in S3 Table. We used iPLEX^TM^ Gold technology (Sequenom Inc., San Diego, CA, USA) according to the manufacturer’s instructions to conduct MALDI-TOF MS-based SNP genotyping as previously described [[19](#_ENREF_19)].

**HVS-I analysis**

We amplified four overlapping fragments of the mtDNA HVS-I control region. These fragments ranged from 93 to 130 bp, and the following primer pairs were used for PCR amplification: L15,989 and H16,158; L16,112 and H16,258; L16,190 and H16,322; and L16,268 and H16,420 [[20-22](#_ENREF_20)]. This combination of primers yielded a 392 bp fragment of the HVS-I region (nps 16,009-16,400). The mutations were established according to the revised Cambridge Reference Sequence (rCRS) [[23](#_ENREF_23),[24](#_ENREF_24)]. The PCR ampliﬁcations were performed in a 25 µL reaction volume containing 6.5 µM MgCl_2_, 0.4 µM dNTPs, 0.66 mg/mL BSA, 1 µM each primer, 2.5 µL GeneAmp 10X PCR Buffer, 3 µL DNA extract, and 1.25 U AmpliTaq Gold^TM^. The PCR reactions were run for 40 cycles at 94°C for 45 s, 56°C for 45 s, and 72°C for 45 s. The PCR products were sequenced using Sanger techniques.

**Authentication criteria**

Endogenous ancient human DNA is difficult to authenticate, mainly due to contamination with modern DNA [[25](#_ENREF_25)]. We consequently took all possible precautions to avoid contamination throughout the various steps. From the excavation site to the laboratory, sampling, storing and within laboratory procedures were performed under DNA-free conditions (except for the 31 samples from the first excavation year, see details above). We gathered two to four extracts from at least two distinct samples per human remains for comparison and further analysis. All sequences reported were confirmed using two to three different DNA extracts for each individual. HVS-I ambiguities were resolved after analyzing multiple sequences originating from at least two amplifications per DNA extract. Consequently, all authentic sequences were deduced from the “consensus” among several sequences from multiple amplification products and extracts. Each individual consensus SNP typing (S8 Table) was deduced from two replicates per extract. Moreover, we systematically assessed whether HVS-I sequences and SNP typing specific to given haplogroups were consistent. All personnel in direct or indirect contact with the remains were genotyped to specifically trace eventual contamination (S2 Table). This contamination traceability and the occurrence of deamination in the sequences confirmed the authenticity of the sequences obtained. Furthermore, Gurgy genetic diversity is similar to other published Neolithic gene pools, with 11 haplotypes (over a total of 27) that have never been described to date in modern European populations.

**Additional population samples used in this study**

We collected sequences from all published ancient DNA data dating from the Paleolithic to the Late Neolithic periods. We divided these data into two groups: samples anterior and contemporaneous to Gurgy, with an upper-time boundary of 4,000 BC (Fig. 2), and samples posterior to Gurgy (groups referred to as ‘PRE’ and ‘POST’ prefixes, respectively, hereafter and throughout the main text). We fixed the limit of 4000 BC to observe admixture effects on Gurgy and (dis)continuity from Gurgy to later populations. We further defined three subsets within each group: hunter-gatherers, Neolithic farmers from Central Europe and Neolithic farmers from Southern Europe (‘HG’, ‘Central_F’ and ‘South_F’ suffixes, respectively, S4 Table). In summary, the following abbreviations were used in the text: PRE_HG (N=41), PRE_Central_F (N=147), PRE_South_F (N=56) (Fig. 2), POST_HG (N=30), POST_Central_F (N=28) and POST_South_F (N=49). Table S4 shows the dates published for the samples used either in BC or BP. We did not use the three individuals from Prissé-la-Charrière [[26](#_ENREF_26)] because the attribution of these individuals to one group or another was not clear. In addition, we considered six ancient populations whose sample sizes were at least 20 sequences (for statistical purposes): Derenburg (N=22[[27](#_ENREF_27),[28](#_ENREF_28)]), Halberstadt (N=31 [[27](#_ENREF_27),[29](#_ENREF_29),[30](#_ENREF_30)]), Karsdorf (N=23 [[29](#_ENREF_29),[30](#_ENREF_30)]), Salzmünde (N=32 [[29](#_ENREF_29),[30](#_ENREF_30)]), Los Cascajos (N=27 [[31](#_ENREF_31)]) and Les Treilles (N=29 [[32](#_ENREF_32)]). Moreover, we gathered a database of 20,535 mitochondrial sequences from 78 modern European and Near East populations (S5 Table). Only available sequences spanning nps 16,024 - 16,380 were retained. The populations were distinguished according to ethnicity, language, culture and geography.

***F*_ST_ and Multidimensional Scaling (MDS) Analyses**

Population-specific pairwise genetic distances (*F*_ST_) were calculated with Arlequin software version 3.5.1.2 [[33](#_ENREF_33)] using a Kimura 2 parameter model [[34](#_ENREF_34)] and a gamma distribution of 0.205 [[35](#_ENREF_35)] (S10 Table). We used the software R version 3.1.2 (Pumpkin Helmet) to perform two-dimensional MDS to display *F*_ST_ values between Gurgy and the ancient dataset on one hand (Fig. 2) and the modern dataset on the other hand (S4 Figure).

**Principal Component Analysis (PCA)**

We performed classical PCAs from haplogroup frequencies using R version 3.1.2 (Pumpkin Helmet). Because haplogroup frequencies were not available for every modern population, we constituted a specific database of 14,304 individuals from 58 modern populations (S5 Table). We also used the six ancient groups and the seven ancient populations cited above. We considered the major European and Near Eastern haplogroups, *i.e*., H, HV, V, J, T, K, N*, N1a, X, I, W, U2, U4, U5a, U5b, and other U. We also considered East Asian (A, B, C, D, F, G and Z) and African haplogroups (L and M1) and an additional group containing all other rare haplogroups (Fig. 1, S5 Figure).

**Median-joining network**

A median-joining network was constructed using the mitochondrial sequences anterior to 4,000 BC for nps 16,056-16,380 with NETWORK 4.6.1.3. (2004-2015 Fluxus Technology Ltd.). Sequences of short lengths (noted with ** on S4 Table) were removed.

**Shared Haplotype Analysis**

We built a new modern database to calculate the percentage of shared haplotypes between Gurgy and modern European populations. We created 31 groups ranging from 400 to 800 samples, with a mean of 622 (S7 Table). Because Bulgarian [[36](#_ENREF_36)] and Scottish [[37](#_ENREF_37)] population samples were too large compared with the other samples, we randomly selected 1/3 and 1/4 of the sequences, respectively (marked with an asterisk on the S11 Table). These sets were distinguished according to ethnicity, language, culture and geography. The percentage of shared informative haplotype was calculated, and a map was generated using Surfer® 12 software (Golden Software, Inc.).

**References**

1. Rottier S, Mordant C, Chambon P, Thevenet C. Découverte de plus d'une centaine de sépultures du Néolithique moyen à Gurgy, les Noisats (Yonne). Bulletin de la Société préhistorique française. 2005; 102: 641-645.

2. Sénépart I. Premiers bergers et paysans des côtes méditerranéennes (5800-4500). In: Demoule J-P, editor. La révolution néolithique en France. Paris: La découverte; 2007. pp. 26-41.

3. Whittle AW. Europe in the Neolithic: the creation of new worlds: Cambridge University Press; 1996. 443 p.

4. Demoule J-P. De l'Europe centrale au Bassin parisien (5200-4400). In: Demoule J-P, editor. La révolution néolithique en France. Paris: La découverte; 2007. pp. 42-59.

5. Manen C, Mazurie De Keroualin K. Les concepts «La Hoguette» et «Limbourg»: un bilan des données. In: Besse M, Stahl Gretsch L-I, Curdy P, editors. ConstellaSion, Hommage à Alain Gallay. Lausanne: Cahiers d'archéologie romande 95; 2003.

6. Jeunesse C. Pratiques funéraires au néolithique ancien: sépultures et nécropoles des sociétés danubiennes (5500/-4900 av. J.-C.): Editions Errance; 1997.

7. Dubouloz J. Datation absolue du premier Néolithique du Bassin parisien: complément et relecture des données RRBP et VSG. Bulletin de la Société préhistorique française. 2003; 100: 671-689.

8. Constantin C, Ilett M. Une étape finale dans le Rubané récent du Bassin parisien. In: Jeunesse C, editor. Le Néolithique danubien et ses marge entre Rhin et Seine. Actes du 22e Colloque interrégional sur le Néolithique (27-29 octobre 1995, Strasbourg); 1997. pp. 207-300.

9. Thevenet C. Une relecture des pratiques funéraires du Rubané récent et final du Bassin parisien: l'exemple des fosses sépulcrales dans la vallée de l'Aisne. Bulletin de la Société préhistorique française. 2004; 101: 815-826.

10. Constantin C, Ilett M. Culture de Blicquy-Villeneuve-Saint-Germain, rapports chronologiques avec les cultures rhénanes. Anthropologie et préhistoire. 1998; 109: 207-216.

11. Jeunesse C. Les groupes régionaux occidentaux du Rubané (Rhin et Bassin parisien) à travers les pratiques funéraires. Gallia préhistoire. 1995; 37: 115-154.

12. Chambon P. Revoir Passy à la lumière de Balloy: les nécropoles monumentales Cerny du bassin Seine-Yonne. Bulletin de la Société préhistorique française. 2003: 505-515.

13. Duhamel P, Mordant D. Les nécropoles monumentales Cerny du bassin Seine-Yonne. Mémoires du Musée de préhistoire d'Ile-de-France. 1997: 481-488.

14. Simonin D, Bach S, Richard G, Vintrou J. Les sépultures sous dalle de type Malesherbes et la nécropole d'Orville. Mémoires du Musée de préhistoire d'Ile-de-France. 1997: 341-379.

15. Thomas A. Identités funéraires, variants biologiques et facteurs chronologiques : une nouvelle perception de contexte culturel et social du Cerny (Bassin parisien, 4700-4300 avant J.-C.): PhD thesis, Bordeaux 1. 2011. 788 p.

16. Demoule J-P, Dubouloz J, Manolakakis L. L'émergence des premières sociétés complexes (4500-3500). In: Demoule J-P, editor. La révolution néolithique en France. Paris: La découverte; 2007. pp. 60-77.

17. Rottier S. L'architecture funéraire des sépultures du Néolithique moyen des Noisats à Gurgy (Yonne, France). Les cistes de Chamblandes et la place des coffres dans les pratiques funéraires du Néolithique moyen occidental. 2007: 99-107.

18. Chambon P (2007) Des Chamblandes au centre de la France ? In: Moinat P, Chambon P, editors. Les cistes de Chamblandes et la place des coffres dans les pratiques funéraires du Néolithique moyen occidental. Actes du colloques de Lausanne, 12 et 13 mai 2006: Cahiers d'archéologie romande 110 et Société préhistorique française XLIII. pp. 75-89.

19. Mendisco F, Keyser C, Hollard C, Seldes V, Nielsen AE, et al. Application of the iPLEX^TM^ Gold SNP genotyping method for the analysis of Amerindian ancient DNA samples: Benefits for ancient population studies. Electrophoresis. 2011; 32: 386-393.

20. Gabriel MN, Huffine EF, Ryan JH, Holland MM, Parsons TJ. Improved MtDNA sequence analysis of forensic remains using a" mini-primer set" amplification strategy. Journal of Forensic Sciences. 2001: 247-253.

21. Adachi N, Shinoda Ki, Umetsu K, Matsumura H. Mitochondrial DNA analysis of Jomon skeletons from the Funadomari site, Hokkaido, and its implication for the origins of Native American. American journal of physical anthropology. 2009; 138: 255-265.

22. Kalmár T, Bachrati CZ, Marcsik A, Raskó I. A simple and efficient method for PCR amplifiable DNA extraction from ancient bones. Nucleic Acids Research. 2000; 28: e67.

23. Anderson S, Bankier AT, Barrell BG, De Bruijn M, Coulson AR, et al. Sequence and organization of the human mitochondrial genome. 1981.

24. Andrews RM, Kubacka I, Chinnery PF, Lightowlers RN, Turnbull DM, et al. Reanalysis and revision of the Cambridge reference sequence for human mitochondrial DNA. Nature genetics. 1999; 23: 147-147.

25. Cooper A, Poinar HN. Ancient DNA: Do It Right or Not at All. Science. 2000; 289: 1139.

26. Deguilloux M-F, Soler L, Pemonge M-H, Scarre C, Joussaume R, et al. News from the west: Ancient DNA from a French megalithic burial chamber. American Journal of Physical Anthropology. 2010; 144: 108-118.

27. Haak W, Forster P, Bramanti B, Matsumura S, Brandt G, et al. Ancient DNA from the First European Farmers in 7500-Year-Old Neolithic Sites. Science. 2005; 310: 1016-1018.

28. Haak W, Balanovsky O, Sanchez JJ, Koshel S, Zaporozhchenko V, et al. Ancient DNA from European early neolithic farmers reveals their near eastern affinities. PLoS biology. 2010; 8: e1000536.

29. Brotherton P, Haak W, Templeton J, Brandt G, Soubrier J, et al. Neolithic mitochondrial haplogroup H genomes and the genetic origins of Europeans. Nature communications. 2013; 4: 1764.

30. Brandt G, Haak W, Adler CJ, Roth C, Szécsényi-Nagy A, et al. Ancient DNA Reveals Key Stages in the Formation of Central European Mitochondrial Genetic Diversity. Science. 2013; 342: 257-261.

31. Hervella M, Izagirre N, Alonso S, Fregel R, Alonso A, et al. Ancient DNA from hunter-gatherer and farmer groups from Northern Spain supports a random dispersion model for the Neolithic expansion into Europe. PLoS One. 2012; 7: e34417.

32. Lacan M, Keyser C, Ricaut F-X, Brucato N, Duranthon F, et al. Ancient DNA reveals male diffusion through the Neolithic Mediterranean route. Proceedings of the National Academy of Sciences. 2011; 108: 9788-9791.

33. Excoffier L, Laval G, Schneider S. Arlequin (version 3.0): an integrated software package for population genetics data analysis. Evolutionary Bioinformatics Online. 2005; 1: 47.

34. Kimura M. A simple method for estimating evolutionary rates of base substitutions through comparative studies of nucleotide sequences. Journal of Molecular Evolution. 1980; 16: 111-120.

35. Ho SY, Endicott P. The crucial role of calibration in molecular date estimates for the peopling of the Americas. American Journal of Human Genetics. 2008; 83: 142-146.

36. Karachanak S, Carossa V, Nesheva D, Olivieri A, Pala M, et al. Bulgarians vs the other European populations: a mitochondrial DNA perspective. International Journal of Legal Medicine. 2012; 126: 497-503.

37. Helgason A, Hickey E, Goodacre S, Bosnes V, Stefánsson K, et al. mtDNA and the islands of the North Atlantic: estimating the proportions of Norse and Gaelic ancestry. The American Journal of Human Genetics. 2001; 68: 723-737.
